# A Systematic Review of Outcomes for People With Intellectual Disabilities and/or Autistic People Following Resettlement From Long‐Stay Hospitals in the UK

**DOI:** 10.1111/jar.70244

**Published:** 2026-05-12

**Authors:** Zoe Rubenstein, Jon Glasby, Rachel Posaner, Robin Miller, Anne‐Marie Glasby

**Affiliations:** ^1^ Department of Social Work and Social Care University of Birmingham Birmingham UK; ^2^ Health Services Management Centre University of Birmingham Birmingham UK; ^3^ Changing Our Lives Crawley UK

**Keywords:** autism, community care, intellectual disabilities, long‐stay hospitals, resettlement, systematic review

## Abstract

**Background:**

Despite attempts to reduce the use of modern secure specialist hospitals (long‐stay hospitals) for people with intellectual disabilities and/or autistic people, there remains a limited understanding of what happens after discharge.

**Method:**

Systematic review of outcomes following resettlement from UK long‐stay hospitals.

**Results:**

Outcomes generally improve following resettlement, although some soon level off and rarely reach the level of comparable populations. We still know little about service costs or about how many readmissions/reconvictions to expect given the complexity of people's needs and such rates in other settings. Above all, most studies fail to meaningfully include the voices of people themselves.

**Conclusions:**

Outcomes improve after hospital, but this is not guaranteed and even improved outcomes may still not be good enough. Future research should aim to fill a number of gaps in current knowledge—but in particular must draw much more meaningfully on the lived experience of people and families.

## Lay Summary

- People with intellectual disabilities and/or autistic people are sometimes kept in hospital for a long time, often in places known as ‘long‐stay hospitals’. When people leave long‐stay hospitals to live in the community, they usually need support to make sure that they can live an ordinary life. We do not know much about what kind of support helps people to live better lives.
- We looked at research that has come out since 1994 to find out what is already known about this issue in the UK. We found that people's lives often improve when they leave long‐stay hospitals, but not always, and they do not improve as much as they could. We do not know much about how much community care costs, or how often people go back to hospital or end up in prison after moving to the community. Most of the studies we read did not include the voices of people themselves.
- We suggest that further research is needed in order to understand what life looks like for people after they leave long‐stay hospitals, and it should include the voices of people themselves.

## 1 Introduction

Research over the past two decades has drawn attention to the number of people with intellectual disabilities and/or autistic people who find themselves ‘stuck’ in secure hospital settings, often for many years and sometimes with no clinical need to remain there (Gibson et al. 2023; Glasby et al. 2024). However, despite significant focus on supporting as many people as possible to leave such settings, we still know very little about what happens next to people and about the sorts of supports that help people to lead more ordinary lives.

The practise of routinely segregating people with intellectual disabilities in large long‐stay hospitals or ‘asylums’ in the UK and internationally had been on the decline since the 1950s, with the last of the asylums closing in the 2000s. While the principles of such a shift from hospital to community were increasingly widely accepted in that time period, there remained concerns about what would happen to some former hospital ‘patients’ in the community, prompting a series of research studies in the 1980s and 1990s to understand longer‐term outcomes.

This research, summarised in a review by Emerson and Hatton (1994), found that most, but not all, people who moved from hospital to the community experienced an improvement in their quality of life (QoL). They also found that improvements varied greatly between services, with some residents experiencing little change in quality of support and most people still experiencing problems in terms of isolation and a lack of choice and control even after moving to the community. Other studies found that improvements levelled off after a few years (Cullen et al. 1995; Locker et al. 1984). Crucially, only a minority of studies identified by Emerson and Hatton (1994) took into account the perspectives of people themselves. At the same time, the shift from asylums to the community has not always equated with increased freedom, as many people continue to experience significant restrictions, especially under Deprivation of Liberty Safeguards (Series 2022).

Despite the closure of the previous asylums, a number of hospital‐based services for people with intellectual disabilities and/or autistic people remained. The historic asylums were large, overcrowded, formidable in design, and based on the principle of containment. These newer forms of provision are much smaller and sometimes (but not always) based in the private sector. They are clinical settings, consisting of highly restrictive environments, and people staying there are typically detained under the Mental Health Act.

Some of these forms of provision are intended to provide short‐term assessment and treatment for people who might represent a danger to themselves or others. In reality, many people are held there because of a lack of appropriate support or housing in the community, sometimes for many years, with no sense of when they might be able to leave. We have elsewhere noted how in practise these hospitals can reproduce some of the elements of the historic asylums, and described this as a process of ‘re‐institutionalisation’ (Glasby et al. 2025). We therefore use the term ‘long‐stay hospital’ to refer to a range of different service models:

*We consider long‐stay hospital settings to be specialist facilities registered as hospitals that are operated by either an NHS or independent sector provider, providing mental or behavioural health‐care in the UK for people with a learning disability or autism. This could be at any level of security (general/low/medium/high), and for people with any status under the Mental Health Act (i.e., admitted informally or detained).* (Ince et al. 2022, p. e3479; originally adapted from NHS Digital).

When the initial asylums closed there were concerns raised about the lack of provision for people with intellectual disabilities and/or autistic people who have mental health diagnoses and/or with the label of ‘behaviour that challenges’ services, for whom community‐based services may not routinely cater (Archer and Watson 1994; Piachaud 1999). People with intellectual disabilities and/or autistic people are also detained in mainstream, non‐specialist mental health hospitals, and some argue that such settings are ill‐equipped to cater to the needs of this population (Melvin et al. 2022). Some in the sector, therefore, regard specialist inpatient provision as useful crisis support (Murphy and Fernando 1999), and there is evidence that inpatient admission can improve outcomes for some people (Melvin et al. 2022; Xenitidis et al. 2004).

However, others fear that such provision puts people at risk of institutional abuse, frequently serves no therapeutic purpose (Mackenzie‐Davies and Mansell 2007; Mansell 2006), and often sees people placed far away from their family and support networks (Yacoub et al. 2008). This argument entered the public domain after a TV documentary exposed the cruel and abusive treatment to which residents of one Assessment and Treatment Unit, Winterbourne View, were routinely subjected. In response to the outcry that followed the revelations, the Department of Health published *Transforming Care* (Department of Health 2012) and *Building the Right Support* (NHS England 2015), with the intention to significantly reduce the use of contemporary long‐stay hospitals.

Despite longstanding official pledges to the contrary, there has been a widespread lack of progress (Cahill et al. 2024). Some 2050 people with intellectual disabilities and/or autistic people were inpatients of hospitals delivering mental or behavioural healthcare in England at the end of December 2024, 52% of whom had a total length of stay of over 2 years, and 16% over 10 years (NHS Digital 2025). There are similar challenges in all four nations of the UK (see, e.g., Ince et al. 2022; Palmer, Boyle, and Wood 2014; Palmer, Rees, and Hatton 2014; Mills et al. 2020; Mental Welfare Commission for Scotland 2016), and similar debates internationally (Davar and Carroll 2024). Resolving such a situation—where people may have multiple needs that span traditional service boundaries and which can involve significant degrees of risk and very expensive service packages—is undoubtedly difficult. However, there are strong moral arguments for facing such a challenge, and evidence that overcoming the barriers is possible (Glasby et al. 2024).

In 2024, Glasby et al. conducted national research to understand the barriers to people with intellectual disabilities and/or autistic people leaving hospital. However, health and social care staff and services in this study were clear that coming out of hospital is the start rather than the end of the process, and that there is no systematic knowledge on what happens next. While commissioners might have some insights, they often change role and commissioning functions are regularly reorganised, meaning that they know little about what happens next. As in our original study, the only people who know what happens after hospital are people themselves and those close to them.

The issue of delayed hospital discharge is entwined with the adequacy of community support. Not only is it important to understand the extent to which inadequate community support may contribute to people being readmitted to hospital, many people cannot be discharged in the first place because there is not enough confidence among decision‐makers as to what works in the community. From our previous research, we now understand the key barriers to people being discharged from long‐stay hospitals after they are clinically ready to leave. Little is known, however, about what allows people to live well in the community.

The last systematic review of outcomes following discharge from long‐stay hospital (Emerson and Hatton 1994) was conducted during the closure of the previous asylums, and long before Transforming Care. To update this review and to explore similar issues in the context of the current landscape, we conducted a systematic literature review with a view to answering the following questions:

1. What happens to people with intellectual disabilities and/or autistic people after leaving ‘long‐stay’ hospitals?
2. What supports help people to have a good QoL and to remain as independent as possible?
3. How do people's QoL and networks change over time after they leave hospital, what does this cost and how does it compare to the costs of being in hospital?

## 2 Methods

For this systematic review we used an integrative method which facilitates the synthesis of insights from quantitative and qualitative data. We began by conducting a systematic search of published and grey literature to identify relevant studies.

### 2.1 Search Strategy

**FIGURE 1:**
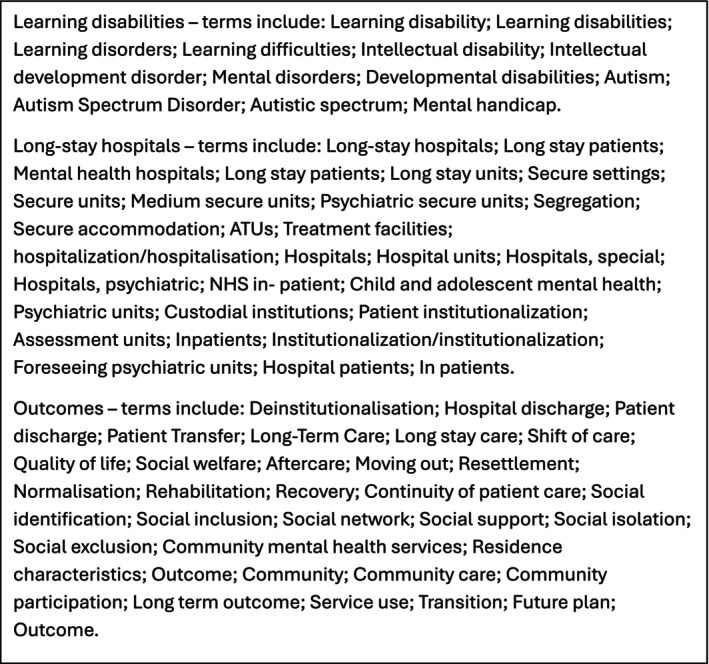
Sample search terms.

As we were interested in what happens to people with intellectual disabilities and/or autistic people after they are discharged from ‘long‐stay’ hospitals, we based our search on a strategy developed by Ince et al. (2022), on which JG, RM and AMG were co‐authors, and which examined delayed long‐stay hospital discharge for people with intellectual disabilities and/or autistic people. We devised a new search strategy which included search terms from Ince et al. (2022) relating to *intellectual disabilities* and *long‐stay hospitals*, but not *delayed discharge*, and added a third category of search terms relating to *outcomes*. Figure 1 shows the search terms which RP used to devise search strategies for different databases. We have made the full search strategies available as Supporting Information.

**FIGURE 2:**
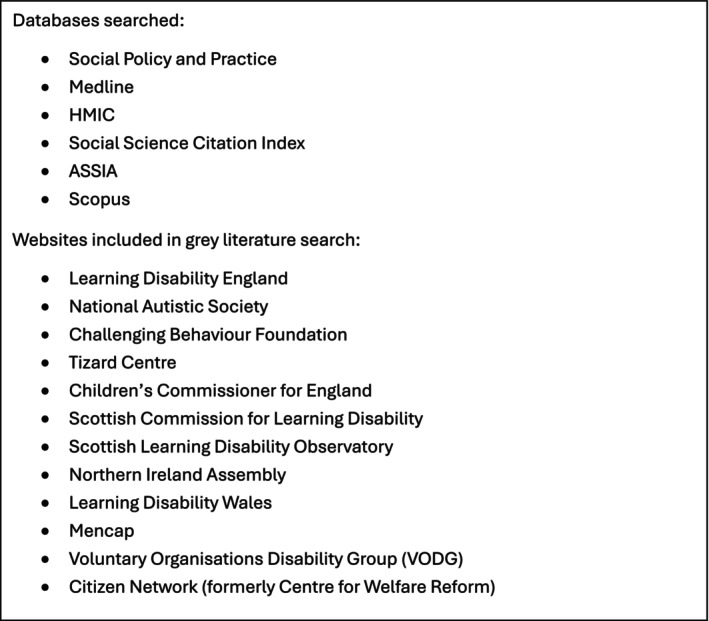
Databases and websites searched. Due to resource constraints, the size of Scopus and difficulty isolating relevant cases, the search strategy within Scopus was targeted [title/proximity].

As the UK context is unique (with further variation among the four nations), and Emerson and Hatton (1994) covered studies conducted before 1994, we limited our search to studies conducted in the UK, published from 1994 onwards. Using these parameters and the above search terms, we searched academic databases and conducted a grey literature search using Google, and on specific government, third sector and research websites. Databases and websites searched are listed in Figure 2.

### 2.2 Screening

**FIGURE 3:**
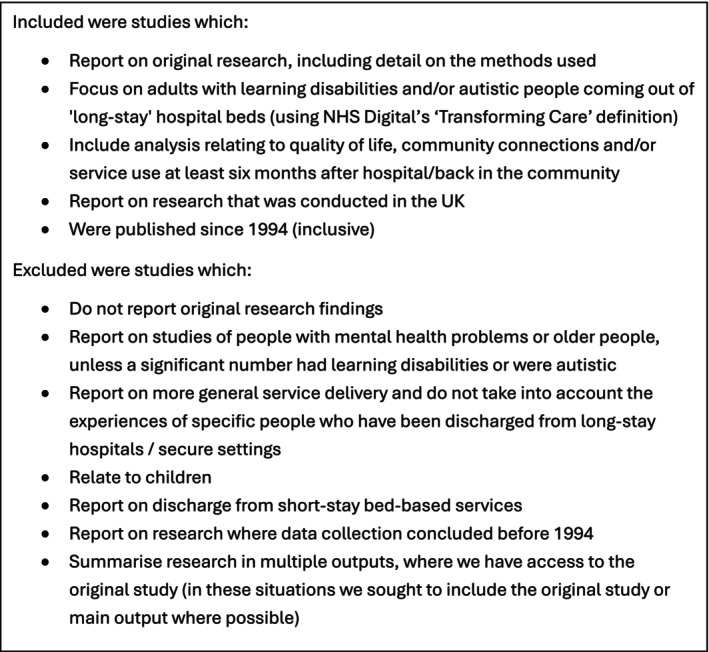
Inclusion and exclusion criteria.

The above methods generated 6627 results from the database search and 46 results from the grey literature search, of which 24 were duplicates. Using Covidence, ZR and JG independently screened the titles and abstracts, applying the inclusion and exclusion criteria listed in Figure 3.

**FIGURE 4:**
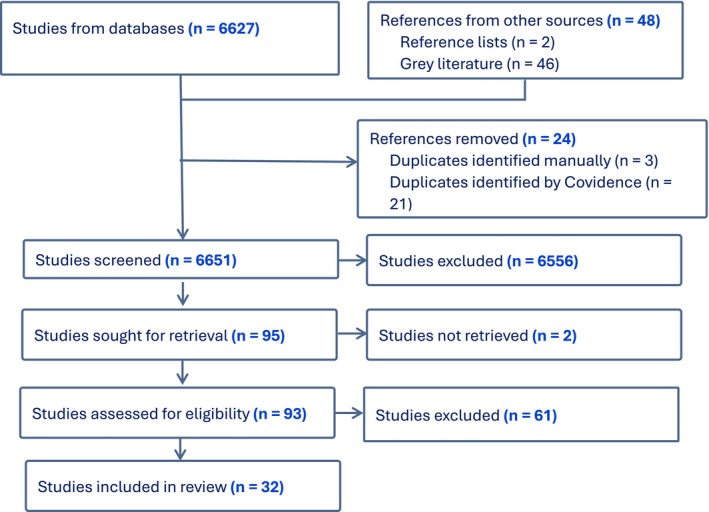
PRISMA flowchart.

Following title/abstract screening, ZR conducted full‐text screening, keeping in regular communication with JG to discuss ambiguities and seek clarity on the inclusion and exclusion criteria. Finally, we screened the reference lists of included studies. This sometimes had the paradoxical impact of *reducing* the number of included studies, for example, where multiple reports from one study cited an original, main output which contained all of the results. Accordingly, we identified 4 further studies through screening reference lists, making a total of 95 studies sought for retrieval; we could not retrieve 2, so full‐text screened 93. Of these, we excluded 61 (including 6 which became duplicates after adding studies from reference lists) and were left with 32 from which to extract data. This process is illustrated in a PRISMA flowchart (Figure 4).

### 2.3 Data Extraction

Data extraction proceeded in two iterative stages. For the first, pilot stage, we developed a data extraction template with which to gather data relating to the study design, findings and our own reflections, and extracted the data using Covidence. We then imported the data into NVivo to check for usability, and used the coding function to develop a more precise, reproducible data extraction template (see Supporting Information) for data relating to study design. For the second stage of data extraction, we used this template in Excel to extract data relating to study design, and imported the studies into NVivo to extract data relating to findings, coding all original and relevant content under the following headings:

- Outcomes:
  ○ Quality of life
  ○ Networks
  ○ Service use
  ○ Costs
  ○ Other
- Discharge planning
- Influencing factors (including success factors and barriers)

Bearing in mind the inconsistencies in reporting across academic texts, we did not limit extraction to either raw data or findings. We also did not conduct a systematic quality appraisal, which would have required identifying a number of tools to fit the wide range of studies identified and would have been unwieldy. Instead, we extracted *any* relevant data or findings and paid attention to whether findings were backed up by the available evidence within the text. Where the justification for claims was clear, but we had questions around the quality of the research or robustness of the claims, we made a note of this and refer to it when reporting the findings. Where claims were made for which we could not find justification in the text, we excluded the claims from synthesis.

### 2.4 Analysis

Once we had completed data extraction using the above‐described method, we proceeded to analyse the data inductively. Using NVivo, we reviewed all the extracted data to identify themes, which facilitated the synthesis of quantitative and qualitative data. We adopted a mixed deductive/inductive approach to analysis. For the deductive element, we retained the headings listed above (outcomes, discharge planning and influencing factors) and developed themes under each of these headings, wherever relevant. For the inductive element, we used coding techniques from Charmaz's (2014) constructivist grounded theory. This allowed us to develop themes from the data while taking account of, but not imposing, our own preconceptions and expectations as to what we would find. The specific coding techniques we applied are designed to allow the researcher to find meaning within the data, rather than remaining at a descriptive level (Miller 2022).

These coding techniques were *initial coding* and *focused coding*. ZR led on the coding, discussing findings with JG regularly and refining the coding framework in line with our discussions. For initial coding, we did so closely and quickly, producing a large number of codes, many of which were overlapping or duplicated. Focused coding allowed us to then develop these into a coherent framework by combining the initial codes into more meaningful themes. The process was iterative; we used initial coding for every included report and periodically used focused coding to bring sense and identify emerging patterns before returning to the next reports to do further initial coding. Throughout the process, we checked the original reports to ensure that the patterns we were identifying were in line with the data we had extracted.

## 3 Results

### 3.1 Overview of Studies

This systematic review included 32 outputs from a total of 27 studies, summarised in Table 1. While we tried to include one ‘main’ output per study, it could be difficult to decide whether some outputs were from the same underlying study, necessitating a judgement call by the current research team. Details as to the methods and focus of the studies can be found in Table 1. Of the six studies which relate to people being discharged from contemporary long‐stay hospitals, three were specifically looking at rates of reoffending, readmission or related outcomes following discharge from a forensic placement (Alexander et al. 2006; Halstead et al. 2001; Wooster et al. 2018). Although these are important outcomes, they tell us little about trends in QoL or engagement in community life.

**TABLE 1:** Summary of studies.

Study/citation	Aim(s)	Methods	Sample size	Original residence	Whose voice?^a^	Contact with (all) people reported?^b^	Key findings
Gogarburn and Tornaveen closure: Ager et al. (2001); Myles et al. (2000)	To measure community integration, variations in costs associated with different models of community care, and factors associated with costs 6–9 months after resettlement.	Longitudinal Quantitative	76	Old‐style asylum	People, care staff, family/unpaid carers (unclear what proportion)	For consent only	Overall increases in quality of life and community integration. Costs varied by age and dependency; costs were negatively associated with accommodation size; no relationship found between costs and outcomes
Alexander et al. (2006)	To measure outcomes relating to (possible) offending and relapse among patients discharged between one and 13 years previously.	Longitudinal Quantitative	27	Contemporary long‐stay hospital	Health and social care staff in the community	No	Offending and level of support reduce over time, but problems persist and a significant minority require long‐term intensive support
Baker (2007)	To measure changes in community use from hospital to up to 18 months after resettlement, and analyse outcomes relating to community use.	Longitudinal Quantitative	28	Old‐style asylum	Care staff	No	Community and leisure use increased following resettlement, but remained low compared to the general population.
Care in the community demonstration project: Beadle‐Brown and Forrester‐Jones (2003); Cambridge et al. (2001)	To identify and analyse factors associated with long‐term outcomes and costs of community care over 12 years following discharge.	Longitudinal Quantitative	282	Old‐style asylum	People, care staff, Researchers	Met all at least once	It is possible to provide good quality, cost effective care in the community, but it is not a guarantee, particularly for people with high support needs or in settings which may become institutional over time. Levels of social impairment were high in hospital and in general did not change significantly over time.
Leicestershire hospital discharge: Bhaumik et al. (2009, 2011)	To measure changes in ‘aggressive challenging behaviour’ and quality of life from before discharge to 1 year following resettlement, and reasons for mortality within 2 years of resettlement	Longitudinal Quantitative	51	Old‐style asylum	Health and social care staff in the community, hospital staff, care staff	No	Identified a statistically significant reduction in observed ‘aggressive challenging behaviour’ over the study period and improvement in quality of life after 6 months, with levelling off after 1 year.
Bryan et al. (2000)	To measure changes in nutritional risk from before discharge to 1 year following resettlement.	Longitudinal Quantitative	118	Old‐style asylum	Hospital staff, health and social care staff in the community, care staff	No	Found increases in risks relating to varied diet, unintentional weight changes and overall risks relating to weight.
Collins and Halman (1996)	To measure changes in behaviour that challenges over 2 years following resettlement.	Longitudinal Quantitative	16	Old‐style asylum	Hospital staff, care staff	No	A small reduction in some categories of behaviour that challenges, also a note of ‘extreme caution’ in the use of rating scales to measure behaviour.
Solihull study: Dagnan and Ruddick (1997); Dagnan et al. (1998)	To measure social networks of people an average of 3.3 years after resettlement, and to identify changes in quality of life over 4.4 years following resettlement.	Longitudinal Quantitative	52	Old‐style asylum	People, care staff (participants present, and participation encouraged for the QoL measure)	Met all at least once	Quality of life improved from resettlement to 3.4 years, and levelled off between 3.4 and 4.4 years. Older people have fewer contact with family members
Donnelly et al. 1996	To measure changes in behaviour, skills and self‐perceived quality of life for people over 24 months after resettlement.	Longitudinal Quantitative	109	Old‐style asylum	People, hospital staff, care staff, researchers (on certain measures people were always asked directly)	No	Minimal changes in behaviour, skills and quality of life, but people reported feeling less depressed and more satisfied with their accommodation.
Ellis et al. 2013	Risk audit at least 18 months after resettlement.	Cross‐sectional Quantitative	36	Old‐style asylum	Health and social care staff in the community, care staff	No	Relatively low risk identified overall, with some areas highlighting a need for intervention.
Fernando et al. 1997	To measure changes in behaviour, skills and abilities between 6 months and 2 years following resettlement.	Longitudinal Quantitative	22	Old‐style asylum	Unclear	No	Overall improvement, particularly in communication, skills, social interaction, ‘stereotyped behaviour’ and ‘symbolic activities’ (e.g., imaginative play, presumed to be age‐inappropriate)
Fish and Lobley (2001)	To measure changes in quality of life 12 months after resettlement from residential to community‐based forensic learning disability service	Longitudinal Quantitative	20	Unclear	People; key workers were asked simply to support, except in one case where two key workers independently completed the questionnaire	Met all at least once	Quantitative measure found no change in quality of life. Qualitative element (conducted 2 months after the move, so too soon to meet inclusion criteria for this study) found both negative and positive changes.
Golding et al. (2005)	To measure changes in competence, behaviour, quality of life, engagement and contact with staff up to 9 months after resettlement.	Longitudinal Quantitative	6	Unclear	Care staff, researchers	Met all multiple times	Relocation may be associated with an increase in skills, decrease in observed ‘problem behaviour’, improved quality of life and more contact with staff.
Halstead et al. (2001)	To measure reoffending, relapse, readmission, death and change of residence among people, who had been in hospital at least a year, up to 5 years following resettlement.	Longitudinal Quantitative	35	Contemporary long‐stay hospital	Hospital staff, care staff, researchers	No	People discharged to treatment from special hospitals or prison, with a primary diagnosis of mental illness, or with a lower IQ, tend to have better outcomes at follow‐up.
Head et al. (2018)	To explore how people with learning disabilities experienced the process of moving as part of Transforming Care, up to 2 years following resettlement.	Cross‐sectional Qualitative	11	Contemporary long‐stay hospital	People, care stuff, unpaid carers	Met all multiple times	Identified a strong influence of friends, family and staff on how people experienced the move, and that people developed new storeys about their identity following the move.
Hubert and Hollins (2010)	To examine changes in experiences for a group of men over 6 years following resettlement.	Longitudinal Qualitative	20	Old‐style asylum	Family, researchers	Met all multiple times	Found improvements to the material conditions of people's lives, but they continued to live institutionalised lives, and the quality of their support was dependent on the home manager.
Leyin (2008)	To measure changes in activities in non‐segregated (i.e., not solely for people with learning disabilities) community settings, over 10 years following resettlement.	Longitudinal Quantitative	18	Old‐style asylum	People, care staff (asked staff to complete the questionnaire with residents, but did not track how often this happened)	No	No change identified in frequency of people engaging in community activities, nor in the proportion of which these could be defined as ‘community participation’ (cite)
McConkey et al. (2003)	To gather the views of former hospital patients and their carers on the move and satisfaction with their lives at least 1 year after resettlement.	Cross‐sectional Qualitative	56	Old‐style asylum	People, family/unpaid carers	Unclear—some direct interviews, some self‐completion questionnaires	Living standards had improved, and residents are in general more satisfied than when in hospital.
Murphy et al. (1996)	To measure quality of life for and gather the views of people an average of 4.5 years following resettlement from a ‘specialist unit’.	Cross‐sectional Mixed‐methods	26	Contemporary long‐stay hospital	People, care staff, family/unpaid carers	Met all multiple times	Quality of life was almost the level of the general population, but considerably lower on some subsets. People had positive and negative views about their placements compared to being in hospital.
Niven et al. (2020)	To review the quality of life and levels of activity of people 10 years following resettlement.	Cross‐sectional Mixed‐methods	15	Contemporary long‐stay hospital	People, health and social care staff in the community, care staff, family/unpaid carers (only one person considered able to self‐report)	Met all at least once	Quality of life was lower than the general population, some improvements were seen but there were persistent issues and improvements were not universal.
Obaydi et al. (1995)	To analyse changes in prescribed medication as an indicator of ‘relocation syndrome’ 1 year following resettlement.	Longitudinal Quantitative	34	Old‐style asylum	Researchers (not recorded, but seemingly data gathered from patient records)	No	Found no evidence of prescribed medication or behavioural changes, indicating that participations did not experience ‘relocation syndrome’.
Owen et al. (2008)	To examine the experiences of transition and community life up to a year following resettlement.	Longitudinal Qualitative	11	Old‐style asylum	Researchers	Met all multiple times	People were insufficiently supported during transition, information was not adequately transferred to their new homes, and lives in the community did not significantly improve compared to hospital.
Perry et al. (2011)	To measure changes in quality of care and lifestyle outcomes for people receiving continuing healthcare funding up to a year after resettlement.	Longitudinal Quantitative	19	Old‐style asylum	People, care staff, researchers	For consent only	Found no significant changes in measured outcomes.
Seager et al. (2000)	To measure readmission rates, and reasons for readmissions up to 11 years following discharge.	Cross‐sectional Quantitative	154	Unclear	Care staff, researchers	No	Physical aggression and physical deterioration were the main reasons for readmission, with a non‐significant trend for people with less severe learning disabilities.
Sines et al. (2012)	To measure changes in quality of life over 18 months following resettlement, through a specially designed tool and qualitative interviews.	Longitudinal Mixed‐methods	39	Old‐style asylum	Health and social care staff in the community, care staff, family/unpaid carers	No	Quality of life improved following resettlement.
Leeds hospital closure: Srivastava and Cooke (1999); Read (2004)	To measure outcomes relating to health, behaviour, morale, skills, quality of life, networks and service design, and to examine causes of mortality over 18 months following resettlement.	Longitudinal Mixed‐methods	111	Old‐style asylum	People, hospital staff, care staff, researchers (only one of 12 measures asked people directly)	No	Quality of life improved, but improvements were not universal and there were a few areas of concern. Found a wide range of positive and negative changes, but analysis was limited and therefore patterns were not identified.
Wooster et al. (2018)	To measure readmission and reoffending among people discharged from a forensic learning disability hospital over a 6 year period.	Cross‐sectional Quantitative	40	Contemporary long‐stay hospital	Health and social care staff in the community	No	Twenty per cent of people were readmitted, and 22.2% were reconvicted following discharge.

^a^ We included ‘people’ under ‘Whose voice’ when any attempt was made to engage people themselves. This includes those studies which asked a minority for whom the research methods were accessible, or where they asked key workers to support people to complete questionnaires, but did not track how often this happened.

^b^ For those studies where researchers met only participants who could communicate, and used a proxy for those who could not, this is noted in Table 1 as ‘no’ under ‘Contact with (all) people reported’. Where researchers did not report on people's views, but took effort to get to know them and understand their experiences, we note this as ‘Met all at least once’.

Three studies, then, sought to understand broad outcomes for people discharged from contemporary long‐stay hospitals. Transforming Care was published in 2012. Of these three studies, one was published in 1996 (Murphy et al. 1996), one was conducted in 2017 but looked at discharge that had taken place in 2007 (Niven et al. 2020), and one study took place fully in the context of the Transforming Care agenda (Head et al. 2018). Taking just these three studies, they echo the broader trends identified here. That is, that discharge can be successful, but that services need to cater to individuals, and that problems persist and they can and should be addressed in community services.

In order to understand how many studies took into account the views of people themselves, we kept track of whose voices were included in the research. Bearing in mind some people with intellectual disabilities would find many research methods inaccessible, we also kept track of where researchers met all participants at least once. See Table 1 for an explanation of how we determined these factors. Four studies met all participants at least once, and six met all multiple times. Of these 10 studies, seven included participant voice, whilst of the 17 remaining studies only six asked people themselves to contribute. Even with a low bar for having been considered to include people's voice, over half the studies included did not meet this threshold. This is a major limitation in the majority of the literature, failing to draw meaningfully on the lived experience of people moving out of hospital. We discuss the implications of this below.

Finally, only six studies included family voice, in spite of families being frequently the only consistent people in the lives of people faced with institutionalisation, and often having the best insight into what good care looks like for their loved ones.

### 3.2 Change in Outcomes After Resettlement

The included studies cover a range of different outcomes, recorded and judged in a number of different ways. While this makes it difficult to identify clear‐cut patterns around specific outcomes, the majority of studies nonetheless suggest general improvements when people move to the community (see, e.g., Ager et al. 2001; Baker 2007; Beadle‐Brown and Forrester‐Jones 2003; Bhaumik et al. 2009, 2011; Cambridge et al. 2001; Collins and Halman 1996; Dagnan and Ruddick 1997, Dagnan et al. 1998; Fernando et al. 1997; Golding et al. 2005; Head et al. 2018; Hubert and Hollins 2010; McConkey et al. 2003; Murphy et al. 1996; Myles et al. 2000; Niven et al. 2020; Owen et al. 2008; Perry et al. 2011; Read 2004; Sines et al. 2012).

However, alongside a number of positives, problems persist for many people, either because improvements are minimal and fail to bring previously even worse outcomes to a satisfactory level, or to the level of comparable populations^1^ (Ager et al. 2001; Baker 2007; Beadle‐Brown and Forrester‐Jones 2003; Dagnan and Ruddick 1997; Head et al. 2018; Hubert and Hollins 2010; Leyin 2008; Owen et al. 2008; Sines et al. 2012), or because outcomes for some have been positive while outcomes for others have been neutral or negative (Alexander et al. 2006; Bhaumik et al. 2011; Cambridge et al. 2001; Donnelly et al. 1996; McConkey et al. 2003; Murphy et al. 1996; Niven et al. 2020; Owen et al. 2008). Moreover, improvements are often observed immediately following resettlement and then level off after the first few years (Bhaumik et al. 2009; Bhaumik et al. 2011; Cambridge et al. 2001; Dagnan et al. 1998; Donnelly et al. 1996; Sines et al. 2012). We explore what this might mean in the discussion, but first review the findings relating to specific outcomes.

### 3.3 Quality of Life and Satisfaction

Findings relating to quantitative measures of QoL were extremely heterogeneous. Some studies found improvements (Ager et al. 2001; Bhaumik et al. 2011; Dagnan et al. 1998; Sines et al. 2012; Golding et al. 2005) while others found no change (Donnelly et al. 1996; Fish and Lobley 2001; Perry et al. 2011). Two included papers reported on cross‐sectional studies of QoL, both finding that QoL was lower than the level of the general population, some areas considerably so, and improvements were not universal (McConkey et al. 2003; Murphy et al. 1996).

This picture is somewhat clouded by a broader methodological issue—with a tendency for some studies to adopt an apparently uncritical reliance on proxy respondents (Ager et al. 2001; Bhaumik et al. 2011; Golding et al. 2005; Sines et al. 2012). These tend to find greater increases in QoL than those that described their efforts to ask people directly, stated how many of the sample were unable to participate, and either took measures to triangulate results where people could not self‐report or excluded those individuals from the sample (Donnelly et al. 1996; Fish and Lobley 2001; Perry et al. 2011). This seems to suggest that others may interpret improvements in people's apparent QoL in a more optimistic manner than the people themselves, raising important issues about who gets to decide what constitutes a good QoL (see below for further discussion).

In terms of morale and satisfaction, Cambridge et al. (2001) found that people were moderately satisfied with their lives, and many were very happy with their accommodation compared to hospital. However, satisfaction was not optimal. At the same time as satisfaction with accommodation increased, for example, a significant minority remained unhappy with the level of privacy and choice, or with continued restrictive practices. Similarly, Donnelly et al. (1996) found that most people were happier with their accommodation compared to when in hospital, but a significant minority showed no change or were less satisfied with their accommodation following resettlement.

Some studies found marked improvements for some, with three studies highlighting positive changes in people's sense of individuality and identities following resettlement. While Head et al. (2018) found that admission to a contemporary long‐stay hospital can have a deep, negative impact on a person's sense of self, resettlement was associated with a transition to a new, adult role, or a return to a previous identity that had been suppressed in hospital. Similarly, Hubert and Hollins (2010) and Owen et al. (2008) found that some people were supported to explore their individuality to a greater extent than in hospital.

McConkey et al. (2003) and Murphy et al. (1996) both found that the majority of participants were happier since resettlement, but a minority were not, and some preferred the hospital to their present placement (which included other institutions for a small minority).

### 3.4 Networks

The size, density and integration with the wider community of some people's networks improved to an extent once out of hospital, but this was often limited and by no means universal. For example, Perry et al. (2011) found that people experienced increased family contact and activity after resettlement, while Head et al. (2018) found that people lost certain important relationships as a result of moving, but had the opportunity to gain new ones, or to foster existing relationships with family. However, Cambridge et al. (2001) found that people still generally had smaller and denser than average social networks compared to the general population, while Murphy et al. (1996) found that networks were small compared to a group of unemployed (not necessarily disabled) people. Similarly, Sines et al. (2012) found that relationships and social interaction increased following resettlement, but remained low compared to the potential. In contrast, Donnelly et al. (1996) found no change in social network following resettlement, while Hubert and Hollins (2010) and McConkey et al. (2003) found that people were not being well supported to develop new relationships.

Studies which addressed the extent to which people accessed their broader community tended to focus on community presence and participation (see Bigby and Wiesel (2011) for a discussion of these concepts). Studies suggest that there is an improvement for many, but still not to the levels of comparable populations and that community participation (while better than in hospital) was generally low. Ager et al. (2001) found that a high proportion of people accessed community facilities, but only a few had contact with people outside their immediate network when doing so. Baker (2007) found that community participation increased following resettlement, but remained at a level lower than the general population, while Niven et al. (2020) also found that levels of participation were lower than population expectations. Leyin (2008) found no change in community presence and community participation or amount of time spent active.

Some studies looked at the extent to which people have others in their networks who do not have an intellectual disability. In much of the literature, this is taken as an inherent good; as evidence of community integration. However, there is little indication within the studies that participants agree that this should be a goal for integration, something acknowledged by Cambridge et al. (2001). Bearing this in mind, Dagnan and Ruddick (1997), a cross‐sectional study, found that few people engaged in leisure activities with people without intellectual disabilities, while Dagnan et al. (1998), a longitudinal study with a subset of the same sample, identified an increase in the amount of contact people had with someone without an intellectual disability. In contrast, Sines et al. (2012) found that nine out of 39 participants appeared to have fewer relationships with people without an intellectual disability following resettlement. One study took its lead from the perspective of participants as to what successful integration looks like (Head et al. 2018), finding that participants were dissatisfied with the degree of integration they were achieving, and that being known in their local community was important to some.

### 3.5 Costs

Only two studies (Cambridge et al. 2001; Myles et al. 2000) reported on costs following resettlement, and only Cambridge et al. (2001) compared costs in hospital and the community. They found that costs were generally higher in the community compared to living in hospital. However, the increase in costs was accompanied by an increase in QoL and quality of care, and costs decreased over the study period. Giving costs at 2002–2003 price levels, Cambridge et al. (2001) note that community care exceeded hospital care by a mean of £163 per week after 1 year, falling to a difference of £29 per week at 12 years. Although these costs are over 20 years' old, and from before Transforming Care, the numbers help to illustrate the differences in costs that *were* identified when comparing hospital‐based to community care. We return to the question of costs and the paucity of contemporary data when exploring influencing factors, and again in the discussion.

### 3.6 Other Outcomes

#### 3.6.1 Readmissions and Reconvictions

Four studies (Alexander et al. 2006; Halstead et al. 2001; Seager et al. 2000; Wooster et al. 2018) looked specifically at outcomes relating to readmissions to hospital or reconvictions for people who had come out of forensic services, or related outcomes such as relapse, offending‐like behaviour or contact with the police. Alexander et al. (2006) found that of 64 people discharged from a forensic service over 12 years, 30% (*n* = 19) had had contact with the police, leading to seven cautions and six reconvictions. Halstead et al. (2001) looked at outcomes for people who had had at least 1 year of treatment in a forensic service, discharged over a 7‐year period. They found that although 34% ‘did something that could have been construed as an offence during the follow‐up period’ (p. 18), only one person was reconvicted in that time.

Wooster et al. (2018) also looked at outcomes for patients discharged from a forensic service over a 7‐year period. They found that 20% of those discharged to the community or prison (rather than another hospital) were readmitted in the time period, and 22.2% of those discharged to the community were convicted of offending. Finally, Seager et al. (2000) looked at readmissions to two intellectual disability hospitals over an 18‐year period and found readmission rates at 8.4%. Interpreting such findings is difficult, as lots of people who have been in prison go on to reoffend (the current rate is thought to be around 26% within 1 year of release [Ministry of Justice 2025]), and a number of people discharged from general hospitals are readmitted (in 2019–20, prior to COVID, nearly 1 million people in England were readmitted to hospital within 30 days of discharge [Nuffield Trust 2022]). While any readmission/reconviction may suggest that being in the community has not worked as well as hoped, therefore, the rates in these studies may actually be much lower than in other service settings.

#### 3.6.2 Behaviour

For the longitudinal studies, measured rates of ‘behaviour that challenges’ showed no clear pattern of change, with most studies finding minimal change over time or increases in one area accompanied by decreases in another. Read (2004) found that, although there was variation within specific areas, behaviour that challenges was relatively stable between resettlement and follow‐up, using a standardised assessment. Fernando et al. (1997) found no change in behaviour that challenges, and Obaydi et al. (1995) found no evidence of ‘behavioural disturbances’ in case notes over the study period. Perry et al. (2011) found a more mixed picture, with no changes in observed behaviour that challenges, but a decrease in the scores on a validated tool conducted in interview with care staff.

Some studies identified a reduction in behaviour that challenges, including Bhaumik et al. (2009) and Golding et al. (2005), although the latter finding was not statistically significant. Donnelly et al. (1996) found an improvement in overall behaviour, but an increase in ‘socially unacceptable’ behaviour, and Collins and Halman (1996) identified a reduction in certain categories of behaviour that challenges. However, Collins and Halman (1996) also urge ‘extreme caution’ in relying on rating scales to measure behaviour, a point we return to in the discussion.

#### 3.6.3 Physical and Mental Health

When measuring changes in health over time, some studies used proxy measures, for example medication as a proxy for changes in health or the degree to which health conditions are being well managed, although there was no clear pattern in these results. In this vein, Bhaumik et al. (2009) and Obaydi et al. (1995) each measured changes in medication for psychiatric conditions, and found no change over the study period, while Read (2004) found a reduction in the use of anticonvulsant, antipsychotic and antidepressant medication. In contrast, Bryan et al. (2000) found an increase in nutritional risk, including many people experiencing unintentional weight changes, undernutrition and obesity, and Fernando et al. (1997) found that measures of mobility and continence had decreased over the study period.

#### 3.6.4 Skills

Studies which measured changes in skills following resettlement produced slightly more optimistic results. Cambridge et al. (2001), Fernando et al. (1997) and Golding et al. (2005) found that people's levels of skill in various domains, including planning and organisation, communication, self‐help and domestic skills, increased following resettlement. Donnelly et al. (1996) found that daily living and social skills increased between the first and second year following resettlement, although the improvement was small and not statistically significant. Niven et al. (2020) was a cross‐sectional study so could not measure change over time, but interviews with respondents indicated that they believed the majority of people had gained new skills since resettlement in areas including domestic, personal care and communication skills.

### 3.7 Factors Influencing Resettlement Outcomes

Here, we explore the elements that were found to impact on outcomes following resettlement, identifying a paucity of evidence on personal attributes and tentative findings relating to service design.

#### 3.7.1 Personal Attributes

Multiple studies explored the extent to which individual attributes contributed to or limited the success of resettlement. We have discussed elsewhere (Glasby et al. 2024) how it is important to avoid using individual characteristics as an ‘explanation’ of outcomes, otherwise we run the risk of ‘blaming the victim’ in a way that would not be considered acceptable in other service settings. Here, personal attributes are included among influencing factors to offer a greater understanding of how outcomes vary for different groups of people.

Although this is an important question then, while individual studies sometimes include a consideration of relationships with factors such as age or gender, the variety of relationships found between outcomes and these variables is too broad to draw conclusions from. Outcomes considered in relation to age were: community engagement (Ager et al. 2001); criminal justice system involvement (Alexander et al. 2006; Halstead et al. 2001); companionship (Cambridge et al. 2001); QoL (Bhaumik et al. 2011) and morale (Cambridge et al. 2001). Outcomes examined in relation to gender were: community activity (Leyin 2008); QoL (Bhaumik et al. 2011) and nutritional risk (Bryan et al. 2000). Outcomes considered in relation to diagnosis, degree of intellectual disability or level of dependence were: criminal justice system involvement (Alexander et al. 2006; Halstead et al. 2001; Seager et al. 2000); community access (Baker 2007); daily living skills (Donnelly et al. 1996); social skills (Beadle‐Brown and Forrester‐Jones 2003; Donnelly et al. 1996) and costs (Myles et al. 2000; Cambridge et al. 2001).

Interestingly, there is little meaningful discussion of ethnicity, which seems a potentially significant omission given what we know about the over‐representation of people from particular Black and minority ethnic communities in mental health services (Bignall et al. 2019). There was also a notable absence of discussion of the differences in outcomes for people with intellectual disabilities and autistic people, and those with a combined diagnosis. We return to this limitation below.

#### 3.7.2 Service Design

Some studies provide tentative insights into the features of service provision that might support more positive outcomes, although few studies measured these relationships empirically. Features of service design which were linked to outcomes explored here are: macro‐level service factors; managerial style, training and support; discharge planning; staffing; and type of accommodation.

The ‘macro level’ here refers to factors that operate on a wider scale, for example, in the way that overall services are designed or purchased, rather than individual service models. Sines et al. (2012) attribute improvements in QoL to the local services working together and to policies relating to standards of care being adhered to following resettlement. Hubert and Hollins (2010), meanwhile, highlighted the importance of continuity of staffing in order for staff to properly get to know the people they are supporting. Cambridge et al. (2001) found that costs in the private sector were lower than expected. However, private providers were much less likely to provide annual revenue costs, and so the conclusions drawn from the data (which were based on publicly available fee information) should be treated with caution.

At the level of care home management, Hubert and Hollins (2010) and Owen et al.'s (2008) in‐depth ethnographic studies identified a strong link between management style and the experiences of residents. They noted how improvements in one home appeared to be closely linked to the specific manager, with a positive managerial attitude being reflected in positive experiences for residents. Other studies drew tentative links between positive outcomes and training (Perry et al. 2011), settings which encouraged residents to take initiative with making plans (Donnelly et al. 1996) and having an individually written plan of action (Baker 2007).

Some studies discussed discharge planning, a few drawing links between the quality or form of discharge planning and the experience of resettlement (Halstead et al. 2001; Head et al. 2018; Hubert and Hollins 2010; Niven et al. 2020; Owen et al. 2008). Others described the discharge planning but did not link it to short or long‐term outcomes (Bhaumik et al. 2009, 2011). Only one study generated evidence on the lasting impact of discharge planning on resettlement, identifying a lack of information transfer as a reason for staff being unable to recognise residents as ‘ordinary human beings’ (Owen et al. 2008, 225).

As well as the impact of training and the culture created by local managers, studies noted the influence of attitudes held by staff themselves. Head et al. (2018) identified the impact of support staff and families around the person in determining their experience of the move and how they experienced shifts in their identities. McConkey et al. (2003) found that over half of family members (19 out of 33) listed staff among a list of things they like. Hubert and Hollins (2010) and Owen et al. (2008) both found that some staff retained patronising attitudes left over from institutional life, and this negatively influenced people's experience of resettlement. There was a notable absence of discussion around trauma‐informed care, and no studies found an association between level of support and outcomes.

Regarding accommodation type, although a large proportion of studies stated where people lived after resettlement, few measured links between accommodation and outcomes. Cambridge et al. (2001) found little difference in morale depending on accommodation, but found that people living in supported accommodation were more likely to express positive views about their living situation; people living in hostels had less privacy than others, and people in hostels and residential/nursing homes were more likely to have neutral or negative views of their neighbours.

Finally, Myles et al. (2000) identified that larger residences were associated with lower costs, especially for people in homes for between 6 and 10 people and in homes for 36 or more people. They noted, however, that there was minimal difference between individual flats and small group homes accommodating between three and five residents.

## 4 Discussion and Conclusion

More than a generation on from Emerson and Hatton's (1994) initial review, and following longstanding and fundamental debates about the risks of ‘re‐institutionalisation’ and the need to ‘transform care’, we still have a mixed picture of what happens after hospital for people with intellectual disabilities and/or autistic people. Although we have included insights from 27 different underlying studies, evidence on outcomes is heterogenous, varies in quality and frequently does not incorporate the views of people themselves. Moreover, most of the studies in the current review relate to the impacts of the closure of previous asylums, rather than to more recent service models and debates.

### 4.1 Changes in Outcomes Over Time

Overall, a range of outcomes seems to improve when people leave hospital and live in more community‐based settings. However, this is not guaranteed or universal; some things can improve and others decline, some people can benefit and others not, and some improvements are modest at best. Despite a number of positives, too many people still seem to be experiencing worse outcomes than comparable populations. Moreover, initial progress often seems to level out, and key questions remain as to whether people can leave hospital, experience some positives and then go on to flourish over the longer‐term. Taking a step back, our view is that no one is too disabled to lead an ordinary life—and that we have to be more ambitious as a society than simply to aim at modest improvements compared to hospital.

### 4.2 The Importance of Lived Experience

Particularly significant was the paucity of studies which engaged meaningfully with people with intellectual disabilities and/or autistic people. Services for people with intellectual disabilities have long proclaimed the importance of ‘nothing about us without us’, and this was embedded throughout previous government policy and practise (via, e.g., a landmark government White Paper, *Valuing People* [Department of Health 2001]). However, studies included here seek to judge people's QoL after leaving hospital without working closely and engaging with people themselves to judge what a good outcome looks like. In our previous study (Glasby et al. 2024), we argued that this is not only morally wrong—but also denies the health and social care system access to a form of expertise that might help to produce different outcomes.

### 4.3 Unanswered/Ongoing Questions

Although the studies reviewed here build on the initial insights provided by Emerson and Hatton in 1994, we still know relatively little about a number of ongoing issues. Crucially, as noted above, the majority of studies included relate to the closure of historic asylums, with only three looking at QoL outcomes for people leaving contemporary long‐stay hospitals. Accordingly, we know little about what happens to people leaving hospital in the context of Transforming Care. Based on the above findings, we highlight two areas as ones which warrant further enquiry.

First, research should undertake to explore what elements of support, service design and broader provision help people to lead a more ordinary life, and what elements of support might lead to more negative outcomes. Such research should take into account how people can be supported to develop their networks and engage in community life in ways that match their own needs and desires, and should explore whether improvements experienced can be maintained and increased to the levels of the general population, rather than levelling off after a few years. Crucially, such research should focus on outcomes that matter to people themselves.

Second, we noted above the absence of data on ethnicity in the research. In our previous study, we reflected on the predominantly white UK nature of our sample, wondering whether this is evidence that some communities are not, in fact, over‐represented in such services, or that some people are not getting support from public services (Glasby et al. 2024). It may also reflect the route that people are likely to take to access mental health services, as Black and minority ethnic people are more likely than white people to enter the criminal justice system (Bignall et al. 2019). Further research should therefore explore whether experiences of resettlement are different for Black and minority ethnic people. Further research should also take note of whether outcomes are different for people with intellectual disabilities compared to autistic people, or those with a combined diagnosis.

In our view, future research must be person‐centred and conducted by research teams skilled at working in such service settings. It would need to work at the pace of the individuals taking part, include the experiences of people who do not communicate verbally, take into account the perspectives of families and grapple with potentially complex issues of capacity and consent. Finally, it will need to translate into practical action for policy and practise so that seldom heard voices are amplified and can make a difference to other people's experiences in future.

## Author Contributions

Zoe Rubenstein contributed to the conception, design, analysis and interpretation of the data, the drafting and revising of the manuscript critically for important intellectual content. She has given final approval of the version to be published and agreed to be accountable for all aspects of the work. Jon Glasby contributed to the conception, design, analysis and interpretation of the data and the drafting and revising of the manuscript critically for important intellectual content. He has given final approval of the version to be published and agreed to be accountable for all aspects of the work. Rachel Posaner contributed to conception and design, acquisition of the data and revising the manuscript critically for important intellectual content. She has given final approval of the version to be published and agreed to be accountable for all aspects of the work. Robin Miller contributed to conception, interpretation of the data and revising the manuscript for important intellectual content. He has given final approval of the version to be published and agreed to be accountable for all aspects of the work. Anne‐Marie Glasby contributed to conception, interpretation of the data, and revising the manuscript for important intellectual content. She has given final approval of the version to be published and agreed to be accountable for all aspects of the work.

## Funding

This work was supported by the National Institute for Health and Care Research (NIHR) School for Social Care Research. The views expressed are those of the author(s) and not necessarily those of the NIHR or the Department of Health and Social Care.

## Ethics Statement

The authors have nothing to report.

## Conflicts of Interest

Changing Our Lives is a rights‐based organisation that is sometimes funded by the NHS to work with people in long‐stay hospital settings to help them and the staff who support them plan what they want their support to look like after hospital.

## Supporting information


**Data S1:** Supporting information.

**Data S2:** Supporting information.

## Data Availability

Data sharing not applicable to this article as no datasets were generated or analysed during the current study.

## Endnotes

^1^
‘Comparable populations’ included people with intellectual disabilities who had not lived in long‐stay hospitals (Baker 2007) and people without paid employment in the general population (Murphy et al. 1996).
