# Supplementary material for: A Systematic Review of Outcomes for People With Intellectual Disabilities and/or Autistic People Following Resettlement From Long‐Stay Hospitals in the UK
Source: J Appl Res Intellect Disabil. 2026 May 12;39:e70244. doi: 10.1111/jar.70244 (PMC13164828; doi:10.1111/jar.70244)
Supplement: Supplementary file 2 — Data S2: Supporting information. [file JAR-39-e70244-s001.docx]

**Your literature search requests from KES**

**Name of literature searcher: Rachel Posaner**

**Date: February 2025**

**Contacts**: Rachel Posaner, [r.d.posaner@bham.ac.uk](mailto:r.d.posaner@bham.ac.uk), Christian Bohm, [c.bohm@bham.ac.uk](mailto:c.bohm@bham.ac.uk) and Rita Perry, [r.perry.1@bham.ac.uk](mailto:r.perry.1@bham.ac.uk)

| **Title of database and dates covered** | **Database Provider** | **Date search conducted and dates covered** | **Notes** | **Results** |
| --- | --- | --- | --- | --- |
| Social Policy & Practice | Ovid | 1994 -  [11/03/2025] | No UK limit, title/abstract and index terms and title for proximity searches.  00Final SPP 06032025 - Why are we stuck | 994 |
| Medline | Ovid | 1994 -  [11/03/2025] | Limit to UK, Index term, title and keyword and title for proximity searches.  00Final Medline 06032025 - Why are we stuck | 1157 |
| HMIC | Ovid | 1994 -  [11/03/2025] | No UK limit, title/abstract and index terms and title for proximity searches.  00Final HMIC 06032025 - Why are we stuck | 1421 |
| Social Science Citation Index |  | 1994 -  [11/03/2025] | Limit to UK, Title/Abstract and Topic*  *Topic =[Title and Author keyword] | 1940 |
| ASSIA |  | 1994 -  [11/03/2025] | Limit to UK, title/abstract and index terms and title for proximity searches  Why are we stuck 11032025 | 2100 |
| Scopus |  | 1994 -  [11/03/2025] | Title/proximity searches | 138 |
| **Results** |  |  |  | **7750** |
| **Duplications** |  |  |  | 1123 |
| **FINAL SEARCH RESULTS (after de-duplication)** |  |  |  | **6627** |

**Search Strategies**

| **Database** | **Search Strategy** |
| --- | --- |
| **Medline** | Database: Ovid MEDLINE(R) ALL <1946 to March 10, 2025> Search Strategy:  --------------------------------------------------------------------------------  1 *Learning Disabilities/ or *Mental Disorders/ or *Autistic Disorder/ or *Intellectual Disability/ or  *Developmental Disabilities/ (235463)  2 ((learning or intellectual* or developmental*) adj3 (disab* or disorder* or difficult* or impair* or severe*)).ti.  (29918)  3 (mental* handicap* or mental disorder* or mental retard* or autistic or autism or asd).ti,kw. (89777)  4 (((challeng* or conduct*) adj3 (disab* or disorder* or difficult* or impair* or behav*)) or (cognitive impair* or  developmental disorder*)).ti. (38763)  5 or/1-4 [Learning Disabilties] (322129)  6 *Hospitals/ or *Hospitals, Psychiatric/ or *Institutionalization/ or *Social Segregation/ (69176)  7 (institution* or "assessment and treatment unit*" or in-patient* or inpatient* or complex care* or segregation or  atu or camh* or hospital*).ti,kw. (1251195)  8 ((long stay* or secure* or custodial* or assessment* or treatment* or special* or mental health* or psychiatric*  or large* or resident*) adj3 (hospital* or unit* or centre* or facilit* or setting or accommodat* or institution* or  patient* or department*)).ti. (120509)  9 or/6-8 [Mental Health Hospitals] (1348286)  10 *Deinstitutionalization/ or deinstitutional*.ti,kw. (1962)  11 *patient discharge/ or *Patient Transfer/ or *Long-Term Care/ or *"Quality of Life"/ or *Social Welfare/ or  *Aftercare/ or *"Continuity of Patient Care"/ or *Social Identification/ or *Social Inclusion/ or *Social Networking/ or *Social Support/ or *Community Mental Health Services/ or *Residence Characteristics/ or (hospital discharge* or  outcome* or social identit* or social isolat* or social exclus* or communit*).ti,kw. (1032048)  12 ((hospital* or patient* or communit*) adj3 (discharge* or transfer* or transition* or plan*)).ti. (22031)  13 ((communit* or social*) adj3 (care* or service* or connnect* or identificat* or inclus* or exclus* or network* or  participat*)).ti. (34605)  14 ("moving out" or resettl* or normalisation* or normalization* or "quality of life" or rehabilit* or recover* or  "long term outcome*" or "service use*" or "social support*" or "social inclusion*" or transition* or "long term care*"  or "long stay care*" or "shift of care*" or "future plan*" or "service transition*" or "social exclusion" or "social identities" or "social identity" or outcome*).ti,kw. (1068632)  15 or/11-14 [ Rehabilitation] (1461360)  16 5 and 9 and 15 (3979)  17 5 and 10 (1042)  18 16 or 17 (4842)  19 (UK or united kingdom or england or ireland or northern ireland or scotland or wales or GB or Great britain).af.  (10272996)  20 18 and 19 (1522)  21 limit 20 to yr="1994 -Current" (1157) |
| **HMIC** | Database: HMIC Health Management Information Consortium <1979 to January 2025> Search Strategy:  --------------------------------------------------------------------------------  1 exp Learning disabilities/ or exp mental disorders/ or exp autism/ or exp autistic spectrum disorders/ or exp  Developmental disorders/ (24401)  2 ((learning or intellectual* or developmental*) adj3 (disab* or disorder* or difficult* or impair* or severe*)).ti.  (2721)  3 (mental* handicap* or mental disorder* or mental retard* or autistic or autism or asd).ti,ab. (4277)  4 (((challeng* or conduct*) adj3 (disab* or disorder* or difficult* or impair* or behav*)) or (cognitive impair* or  developmental disorder*)).ti. (301)  5 or/1-4 [Learning Disabilties] (25824)  6 exp Mental health hospitals/ or exp Learning disability hospitals/ or exp hospitals/ or exp Segregation/ or exp  Patient institutionalisation/ or exp Institutionalisation/ or psychiatric hospital*.ti,ab. (16536)  7 (institution* or "assessment and treatment unit*" or in-patient* or inpatient* or complex care* or segregation or  atu or camh* or hospital*).ti,ab. (62052)  8 ((long stay* or secure* or custodial* or assessment* or treatment* or special* or mental health* or psychiatric*or  large* or resident*) adj3 (hospital* or unit* or centre* or facilit* or setting or accommodat* or institution* or  patient* or department*)).ti. (3033)  9 or/6-8 [Mental Health Hospitals] (68444)  10 deinstitutionalis*.ti,ab. or exp Care in the community/ (224)  11 exp Patient discharge/ or exp Patient transfer/ or exp "Quality of life"/ or exp Social welfare/ or exp After  care/ or exp "Continuity of patient care"/ or exp Outcomes/ or exp Social inclusion/ or exp Social isolation/ or exp Social networking/ or exp Social support/ or exp Social exclusion/ or exp Community mental health services/ or exp Communities/ or (hospital discharge* or social identit*).ti,ab. (29481)  12 ((hospital* or patient* or communit*) adj3 (discharge* or transfer* or transition* or plan*)).ti. (1293)  13 ((communit* or social*) adj3 (care* or service* or connnect* or identificat* or inclus* or exclus* or network* or  participat*)).ti. (15014)  14 ("moving out" or resettl* or normalisation* or normalization* or "quality of life" or rehabilit* or recover* or  "long term outcome*" or "service use*" or "social support*" or "social inclusion*" or transition* or "long term care*or long stay care*" or "shift of care*" or "future plan*" or "service transition*" or "social exclusion" or "social identities" or "social identity" or outcome*).ti,ab. (48271)  15 or/11-14 [ Rehabilitation] (78151)  16 5 and 9 and 15 (2048)  17 5 and 10 (144)  18 16 or 17 (2147)  19 limit 18 to yr="1994 -Current" (1421) |
| **Social Policy & Practice** | Database: Social Policy and Practice <202502> Search Strategy:  --------------------------------------------------------------------------------  1 (learning disabilities or mental disorder or autism or autistic spectrum conditions or autistic spectrum  disorders).de. (18495)  2 ((learning or intellectual* or developmental*) adj3 (disab* or disorder* or difficult* or impair* or severe*)).ti.  (7517)  3 (mental* handicap* or mental disorder* or mental retard* or autistic or autism or asd).ti,ab. (4927)  4 (((challeng* or conduct*) adj3 (disab* or disorder* or difficult* or impair* or behav*)) or (cognitive impair* or  developmental disorder*)).ti. (1322)  5 or/1-4 [Learning Disabilties] (22242)  6 (hospitals or institutionalisation or social segregation or psychiatric hospital or psychiatric hospitals).de.  (4249)  7 (institution* or "assessment and treatment unit*" or in-patient* or inpatient* or complex care* or segregation or  atu or camh* or hospital*).ti,ab. (29167)  8 ((long stay* or secure* or custodial* or assessment* or treatment* or special* or mental health* or psychiatric*or  large* or resident*) adj3 (hospital* or unit* or centre* or facilit* or setting or accommodat* or institution* or  patient* or department*)).ti. (1982)  9 or/6-8 [Mental Health Hospitals] (31468)  10 Deinstitutionalisation.de. or deinstitutional*.ti,ab. (328)  11 (patient discharge or hospital discharge or patient transfer or quality of life or social welfare or aftercare or  continuity of care or outcomes or social identities or social identity or social inclusion or social isolation or social networking or social support or social exclusion or community mental health services or communities).de. (51545)  12 ((hospital* or patient* or communit*) adj3 (discharge* or transfer* or transition* or plan*)).ti. (1187)  13 ((communit* or social*) adj3 (care* or service* or connnect* or identificat* or inclus* or exclus* or network* or  participat*)).ti. (23396)  14 ("moving out" or resettl* or normalisation* or normalization* or "quality of life" or rehabilit* or recover* or  "long term outcome*" or "service use*" or "social support*" or "social inclusion*" or transition* or "long term care*or long stay care*" or "shift of care*" or "future plan*" or "service transition*" or "social exclusion" or "social identities" or "social identity" or outcome*).ti,ab. (79456)  15 or/11-14 [ Rehabilitation] (122878)  16 5 and 9 and 15 (1005)  17 5 and 10 (159)  18 16 or 17 (1101)  19 limit 18 to yr="1994 -Current" (994) |
| **Social Science Citation Index** | 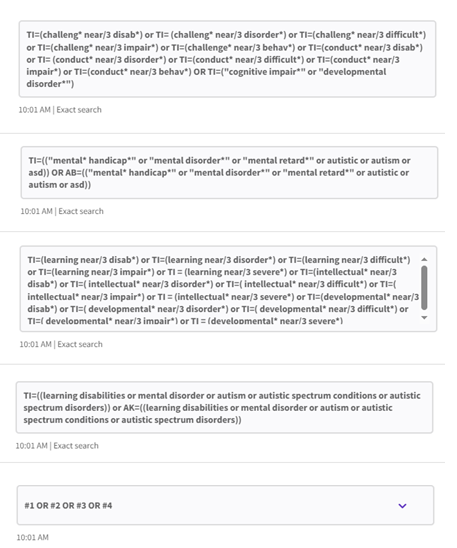  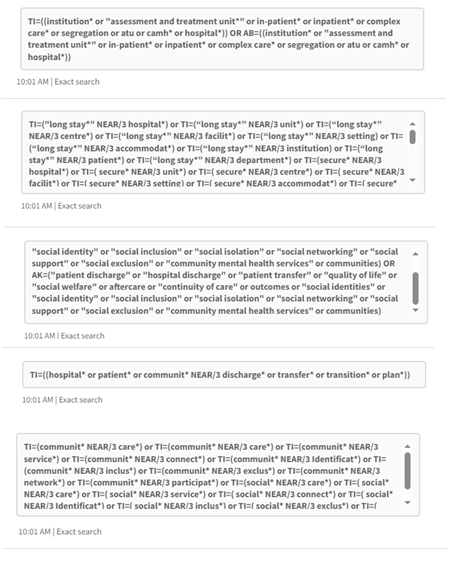  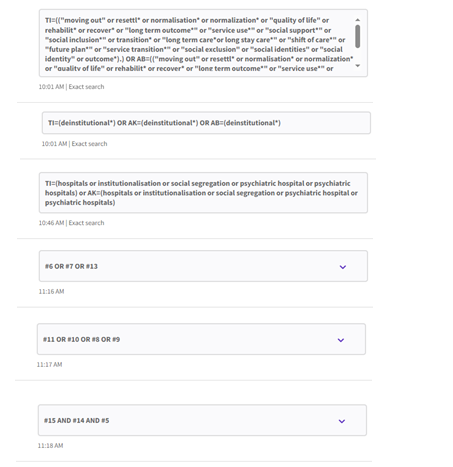  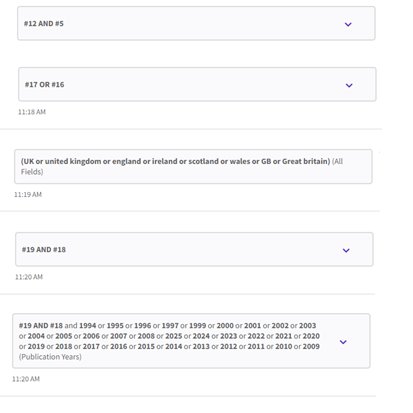 |
| **Scopus** | (ALL ( ( uk OR "united kingdom" OR england OR ireland OR "northern Ireland" OR scotland OR wales OR gb OR "Great Britain" ) )) AND (( ( ( ( TITLE ( ( communit* OR social* ) W/3 ( care* OR service* OR connnect* OR identificat* OR inclus* OR exclus* OR network* OR participat* ) ) OR TITLE ( ( "moving out" OR resettl* OR normalisation* OR normalization* OR "quality of life" OR rehabilit* OR recover* OR "long term outcome*" OR "service use*" OR "social support*" OR "social inclus*" OR "social isolate*" OR transition* OR "long term care*" OR "long stay care*" OR "shift of care*" OR "future plan*" OR "service transition*" OR "social exclus*" OR "social identit*" OR outcome* OR "social welfare*" OR aftercare* OR "continuity of care*" OR "social network*" OR communit* ) ) ) ) AND ( ( TITLE ( ( learning OR intellectual* OR developmental* ) W/3 ( disab* OR disorder* OR difficult* OR impair* OR severe* ) ) OR TITLE ( ( challeng* OR conduct* ) W/3 ( disab* OR disorder* OR difficult* OR impair* OR behav* ) ) OR TITLE ( ( "cognitive impair*" OR "mental* handicap*" OR "mental disorder*" OR "mental retard*" OR autistic OR autism OR asd ) ) ) ) AND ( ( TITLE ( ( "long stay*" OR secure* OR custodial* OR assessment* OR treatment* OR special* OR "mental health*" OR psychiatric* OR large* OR resident* ) W/3 ( hospital* OR unit* OR centre* OR facilit* OR setting OR accommodat* OR institution* OR patient* OR department* ) ) ) OR ( TITLE ( ( institution* OR "assessment and treatment unit*" OR in-patient* OR inpatient* OR complex AND care* OR segregation OR atu OR camh* OR hospital* ) ) ) ) ) OR ( ( TITLE ( deinstitutional* ) ) AND ( ( TITLE ( ( learning OR intellectual* OR developmental* ) W/3 ( disab* OR disorder* OR difficult* OR impair* OR severe* ) ) OR TITLE ( ( challeng* OR conduct* ) W/3 ( disab* OR disorder* OR difficult* OR impair* OR behav* ) ) OR TITLE ( ( "cognitive impair*" OR "mental* handicap*" OR "mental disorder*" OR "mental retard*" OR autistic OR autism OR asd ) ) ) ) ) AND PUBYEAR > 1993 AND PUBYEAR < 2026 ) |
| **ASSIA** | (((((MAINSUBJECT.EXACT("Learning disabilities") OR (MAINSUBJECT.EXACT("Developmental disabilities") OR MAINSUBJECT.EXACT("Developmental disorders") OR MAINSUBJECT.EXACT("Mental disorders")) OR MAINSUBJECT.EXACT("Autism") OR MAINSUBJECT.EXACT("Intellectual disabilities")) OR title((learning NEAR/3 disab*) OR (learning NEAR/3 disorder*) OR (learning NEAR/3 difficult*) OR (learning NEAR/3 impair*) OR (learning NEAR/3 severe*) OR (intellectual* NEAR/3 disab*) OR (intellectual* NEAR/3 disorder*) OR (intellectual* NEAR/3 difficult*) OR (intellectual* NEAR/3 impair*) OR (intellectual* NEAR/3 severe*) OR (developmental* NEAR/3 disab*) OR (developmental* NEAR/3 disorder*) OR (developmental* NEAR/3 difficult*) OR (developmental* NEAR/3 impair*) OR (developmental* NEAR/3 severe*)) OR (title((“mental* handicap*” OR ("mental disorder" OR "mental disorders") OR ("mental retardation") OR autistic OR autism OR asd)) OR abstract((“mental* handicap*” OR ("mental disorder" OR "mental disorders") OR ("mental retardation") OR autistic OR autism OR asd))) OR (title((challeng* NEAR/3 disab*) OR (challeng* NEAR/3 disorder*) OR (challeng* NEAR/3 difficult*) OR (challeng* NEAR/3 impair*) OR (challenge* NEAR/3 behav*) OR (conduct* NEAR/3 disab*) OR (conduct* NEAR/3 disorder*) OR (conduct* NEAR/3 difficult*) OR (conduct* NEAR/3 impair*) OR (conduct* NEAR/3 behav*)) OR title((("cognitive impairment" OR "cognitive impairments") OR ("developmental disorder" OR "developmental disorders"))))) AND (MAINSUBJECT.EXACT("Deinstitutionalization") OR MAINSUBJECT.EXACT("Deinstitutionalized") OR title(deinstitutional*) OR abstract(deinstitutional*))) OR (((MAINSUBJECT.EXACT("Learning disabilities") OR (MAINSUBJECT.EXACT("Developmental disabilities") OR MAINSUBJECT.EXACT("Developmental disorders") OR MAINSUBJECT.EXACT("Mental disorders")) OR MAINSUBJECT.EXACT("Autism") OR MAINSUBJECT.EXACT("Intellectual disabilities")) OR title((learning NEAR/3 disab*) OR (learning NEAR/3 disorder*) OR (learning NEAR/3 difficult*) OR (learning NEAR/3 impair*) OR (learning NEAR/3 severe*) OR (intellectual* NEAR/3 disab*) OR (intellectual* NEAR/3 disorder*) OR (intellectual* NEAR/3 difficult*) OR (intellectual* NEAR/3 impair*) OR (intellectual* NEAR/3 severe*) OR (developmental* NEAR/3 disab*) OR (developmental* NEAR/3 disorder*) OR (developmental* NEAR/3 difficult*) OR (developmental* NEAR/3 impair*) OR (developmental* NEAR/3 severe*)) OR (title((“mental* handicap*” OR ("mental disorder" OR "mental disorders") OR ("mental retardation") OR autistic OR autism OR asd)) OR abstract((mental* handicap* OR mental disorder* OR mental retard* OR autistic OR autism OR asd))) OR (title((challeng* NEAR/3 disab*) OR (challeng* NEAR/3 disorder*) OR (challeng* NEAR/3 difficult*) OR (challeng* NEAR/3 impair*) OR (challenge* NEAR/3 behav*) OR (conduct* NEAR/3 disab*) OR (conduct* NEAR/3 disorder*) OR (conduct* NEAR/3 difficult*) OR (conduct* NEAR/3 impair*) OR (conduct* NEAR/3 behav*)) OR title((("cognitive impairment" OR "cognitive impairments") OR ("developmental disorder" OR "developmental disorders"))))) AND (((MAINSUBJECT.EXACT("Learning disability hospitals") OR MAINSUBJECT.EXACT("Hospitals")) OR MAINSUBJECT.EXACT("Psychiatric hospitals") OR MAINSUBJECT.EXACT("Institutionalization") OR MAINSUBJECT.EXACT("Segregation")) OR (title((institution* OR "assessment and treatment unit*" OR in-patient* OR inpatient* OR ("complex care") OR segregation OR atu OR camh* OR hospital*)) OR abstract((institution* OR "assessment and treatment unit*" OR in-patient* OR inpatient* OR ("complex care") OR segregation OR atu OR camh* OR hospital*))) OR title((("long stay" OR "long stayed" OR "long stays") NEAR/3 hospital*) OR (("long stay" OR "long stayed" OR "long stays") NEAR/3 unit*) OR (("long stay" OR "long stayed" OR "long stays") NEAR/3 centre*) OR (("long stay" OR "long stayed" OR "long stays") NEAR/3 facilit*) OR (("long stay" OR "long stayed" OR "long stays") NEAR/3 setting) OR (("long stay" OR "long stayed" OR "long stays") NEAR/3 accommodat*) OR (("long stay" OR "long stayed" OR "long stays") NEAR/3 institution) OR (("long stay" OR "long stayed" OR "long stays") NEAR/3 patient*) OR (("long stay" OR "long stayed" OR "long stays") NEAR/3 department*) OR (secure* NEAR/3 hospital*) OR (secure* NEAR/3 unit*) OR (secure* NEAR/3 centre*) OR (secure* NEAR/3 facilit*) OR (secure* NEAR/3 setting) OR (secure* NEAR/3 accommodat*) OR (secure* NEAR/3 institution) OR (secure* NEAR/3 patient*) OR (secure* NEAR/3 department*) OR (custodial* NEAR/3 hospital*) OR (custodial* NEAR/3 unit*) OR (custodial* NEAR/3 centre*) OR (custodial* NEAR/3 facilit*) OR (custodial* NEAR/3 setting) OR (custodial* NEAR/3 accommodat*) OR (custodial* NEAR/3 institution) OR (custodial* NEAR/3 patient*) OR (custodial* NEAR/3 department*) OR (assessment* NEAR/3 hospital*) OR (assessment* NEAR/3 unit*) OR (assessment* NEAR/3 centre*) OR (assessment* NEAR/3 facilit*) OR (assessment* NEAR/3 setting) OR (assessment* NEAR/3 accommodat*) OR (assessment* NEAR/3 institution) OR (assessment* NEAR/3 patient*) OR (assessment* NEAR/3 department*) OR (treatment* NEAR/3 hospital*) OR (treatment* NEAR/3 unit*) OR (treatment* NEAR/3 centre*) OR (treatment* NEAR/3 facilit*) OR (treatment* NEAR/3 setting) OR (treatment* NEAR/3 accommodat*) OR (treatment* NEAR/3 institution) OR (treatment* NEAR/3 patient*) OR (treatment* NEAR/3 department*))) AND ((MAINSUBJECT.EXACT("Hospital discharged") OR MAINSUBJECT.EXACT("Quality of life") OR MAINSUBJECT.EXACT("Social welfare") OR MAINSUBJECT.EXACT("After care") OR MAINSUBJECT.EXACT("Outcomes") OR MAINSUBJECT.EXACT("Social identity") OR MAINSUBJECT.EXACT("Social integration") OR MAINSUBJECT.EXACT("Social networks") OR MAINSUBJECT.EXACT("Social support") OR MAINSUBJECT.EXACT("Social exclusion")) OR title((hospital* NEAR/3 discharge*) OR (hospital* NEAR/3 transfer*) OR (hospital* NEAR/3 transition*) OR (hospital* NEAR/3 plan*) OR (patient* NEAR/3 discharge*) OR (patient* NEAR/3 transfer*) OR (patient* NEAR/3 transition*) OR (patient* NEAR/3 plan*) OR (communit* NEAR/3 discharge*) OR (communit* NEAR/3 transfer*) OR (communit* NEAR/3 transition*) OR (communit* NEAR/3 plan*)) OR title((communit* NEAR/3 care*) OR (communit* NEAR/3 service*) OR (communit* NEAR/3 connect*) OR (communit* NEAR/3 identificat*) OR (communit* NEAR/3 inclus*) OR (communit* NEAR/3 exclus*) OR (communit* NEAR/3 network*) OR (communit* NEAR/3 participat*) OR (social* NEAR/3 care*) OR (social* NEAR/3 service*) OR (social* NEAR/3 connect*) OR (social* NEAR/3 indentificat*) OR (social* NEAR/3 inclus*) OR (social* NEAR/3 exclus*) OR (social* NEAR/3 network*) OR (social* NEAR/3 participat*)) OR (title(("moving out" OR resettl* OR normalisation* OR normalization* OR "quality of life" OR rehabilit* OR recover* OR "long term outcome*" OR ("service use" OR "service used" OR "service user" OR "service users" OR "service uses") OR ("social support" OR "social supports") OR ("social inclusion") OR transition* OR "long term care*or long stay care*" OR "shift of care*" OR ("future plan" OR "future planetary" OR "future planned" OR "future planning" OR "future plans" OR "future plants") OR ("service transition") OR "social exclusion" OR "social identities" OR "social identity" OR outcome*)) OR abstract(("moving out" OR resettl* OR normalisation* OR normalization* OR "quality of life" OR rehabilit* OR recover* OR "long term outcome*" OR ("service use" OR "service used" OR "service user" OR "service users" OR "service uses") OR ("social support" OR "social supports") OR ("social inclusion") OR transition* OR "long term care*" or "long stay care*" OR "shift of care*" OR ("future plan" OR "future planetary" OR "future planned" OR "future planning" OR "future plans" OR "future plants") OR ("service transition") OR "social exclusion" OR "social identities" OR "social identity" OR outcome*))) OR (MAINSUBJECT.EXACT("Community mental health services") OR MAINSUBJECT.EXACT("Community") OR title(("patient discharge*" OR ("patient transfer" OR "patient transfers") OR "continuity of care" OR "social insolat*")) OR abstract(("patient discharge*" OR ("patient transfer" OR "patient transfers") OR "continuity of care" OR "social insolat*")))))) AND (noft(UK) OR noft("united kingdom") OR noft(england) OR noft(ireland) or noft("northern ireland") OR noft(scotland) OR noft(wales) OR noft(GB) OR noft("Great britain"))) AND pd(19940101-20250310) |

We hope that you find the evidence search service useful. Whilst care has been taken in the selection of the materials included in this evidence search, KES is not responsible for the content or the accuracy of the enclosed research information. Accordingly, whilst every endeavour has been undertaken to execute a comprehensive search of the literature, KES is not and will not be held responsible or liable for any omissions to pertinent research information not included as part of the results of the enclosed evidence search. Requestors are welcome to discuss the evidence search findings with the librarian responsible for executing the search. We welcome suggestions on additional search strategies / use of other information resources for further exploration. You must not use the results of this search for commercial purposes. Any usage or reproduction of the search output should acknowledge the KES Service that produced it.
